# Effect of sepsis challenge in chronic inflammation state on mortality and long-term pathological findings in rats

**DOI:** 10.1186/cc12972

**Published:** 2013-11-05

**Authors:** Roberto Tussi-Junior, Eduardo LS Bastos, Ana MCRPF Martins, Rodrigo B Souza, Ana MA Liberatore, José CF Vieira, Ivan HJ Koh

**Affiliations:** 1 Department of Surgery, Federal University of São Paulo, Brazil; 2 Institute of Biology of São Paulo State, São Paulo, Brazil; 3 Department of Pediatrics, Federal University of São Paulo, Brazil

## Background

Recent studies from our laboratory showed that animals subjected to 50% shortening of the small intestine developed bacterial translocation unleashed chronically. Bacterial translocation has shown the effect of exacerbation of systemic inflammatory response by crosstalk between intestinal and systemic immune response. In this sense, the aim of this study was to evaluate whether a septic challenge in the state of chronic inflammation resulting from the shortening of the small bowel can modify the mortality outcome and trigger organ alterations in the long term.

## Materials and methods

Wistar-EPM rats were submitted to 50% small intestine shortening (IS group, *n *= 20) or sham intestinal anastomosis (IA group, *n *= 20), and after 4 months were submitted to sepsis challenge with 2 ml 10^8^CFU/ml *Escherichia coli *i.v. The mortality was observed up to 30 days and the survivors of both groups were killed after 6 months for histological analysis. The other 10 animals were killed after 4 months of intestinal shortening in order to determine the histological pattern related to the bowel shortening effect.

## Results

The mortality rate after sepsis was 80% in the IS group and 35% in the IA group. The bowel shortening without sepsis challenge showed hepatic mild steatosis with inflammation similar to acute hepatitis, vascular congestion and focal necrosis. The distal ileum showed shortening and broadening of villus, focal cryptic necrosis and mild macrophages and eosinophil infiltration in the lamina propria. In the IS group was seen a generalized steatosis and vascular congestion in the liver; alveolar atelectasis, BALT hyperplasia, a large number of macrophages, mast cells, foam cells, lymphocytes, eosinophil and plasmocyte infiltration and alveolar edema, plus vascular congestion and sclerosis in the lung; villus apical necrosis, intense inflammatory cell infiltration and vascular congestion in the lamina propria of the ileum; and the kidney with tubular nephrosis, tubular obstruction, vascular congestion with interstitium hemorrhage and tubular hyaline material deposition. In the IA group was seen moderate liver steatosis, intestinal lamina propria cellular infiltrations, glomerulonephritis, kidney tubular edema, parenchymal hemorrhage and Bowman capsule thickness. However, the alterations were less compared with the IS group.

## Conclusions

The chronic inflammatory state, in combination with sepsis, might be an important aggravating factor related to sepsis morbidity and mortality by promoting an increasing organ dysfunction in the long term.

